# Onychomycosis prevalence etiology and associated factors in women using nail cosmetics attending Mbarara regional referral hospital dermatology clinic Uganda

**DOI:** 10.1038/s41598-025-30250-8

**Published:** 2025-12-11

**Authors:** Ronnie Mayengo, Nalumaga Pauline Petra, Oloro Joseph, Edward Ogwang, Grace Mulyowa Kitunzi, Aloyo Gladys Onguti, Stephen Kizito Mirembe

**Affiliations:** 1 Department of Dermatology, Mbarara University of Science and Technology, Mbarara, Uganda; 2 Department of Microbiology, Mbarara University of Science and Technology, Mbarara, Uganda; 3 Department of Pharmacology, Mbarara University of Science and Technology, Mbarara, Uganda; 4 Department of Internal Medicine, Gulu University, Gulu, Uganda

**Keywords:** Onychomycosis, Nail cosmetics, Dermatophytes, Prevalence, Risk factors, Diseases, Health care, Medical research, Microbiology, Risk factors

## Abstract

Onychomycosis is a fungal infection of the nails caused primarily by dermatophytes, Non-Dermatophyte Moulds (NDMs) and yeast species. Fungal transmission occurs through direct contact with contaminated objects. Nail cosmetic treatments involve using tools that may be contaminated with fungus and can traumatize the nail, potentially increasing the risk of infection. However, little is known about its prevalence, etiology and associated factors among women using nail cosmetics in Uganda. A cross-sectional study at Mbarara Regional Referral Hospital (MRRH) skin clinic (January–March 2025) enrolled 273 women with a history of nail cosmetic use. Nail clippings of participants underwent direct microscopy and fungal culture to assess the prevalence and etiology. Fungal isolates were identified morphologically using lactophenol cotton blue staining. Data collected using a structured questionnaire were analyzed in STATA 12.0. Univariate and multivariate logistic regression identified associated factors; adjusted odds ratios (ORs), 95% confidence intervals (CIs), and *p*-values were calculated using Wald’s test (*p* < 0.05 significant). The prevalence of onychomycosis among women using nail cosmetic was 57.5%. Dermatophytes were the most frequently isolated organisms, with *Trichophyton mentagrophytes* being predominant, followed by NDMs and *Candida* species. Frequent nail polish application, nail trauma and sales/retail occupations showed a statistically significant association with onychomycosis (*p* < 0.05). Onychomycosis was prevalent among women using nail cosmetics, with predominance of *Trichophyton mentagrophytes.* And associated with nail trauma, frequent application, and sales/retail occupation. These associations suggest that trauma and frequent application may elevate infection risk, while henna offers potential protection with antifungal properties. These findings highlight the need for public awareness and improved hygiene standards in nail care practices.

## Introduction

Onychomycosis is a chronic fungal infection of the nail unit that compromises nail integrity and is associated with aesthetic concerns, functional impairment, and the risk of secondary infections^1,2^. Globally, onychomycosis is responsible for approximately 50% of all nail disorders, with prevalence estimates ranging from 5.5 to 60% depending on regional climate variations and risk populations such as the elderly, dialysis patients, and occupational exposure^3,4^. In Africa, a meta-analysis of 13 studies reported a pooled prevalence of 19.6%, with significant regional variation of 68.0% in North Africa and 7.7% in East Africa, attributed to differences in healthcare infrastructure, hygiene practices, and environmental conditions^5^.

Globally, onychomycosis is predominantly caused by Dermatophytes, notably *Trichophyton rubrum* and *T. mentagrophytes*, accounting for 75–90% of cases due to their ability to degrade keratin^6–8^. However, non-dermatophyte molds (NDMs) such as *Aspergillus* and *Fusarium* species, alongside yeasts like *Candida albicans*, are increasingly reported, particularly in humid, warm environments that favor their proliferation^9,10^. In East Africa, *Candida albicans* has emerged as a significant contributor, reflecting regional microbiological patterns^5^.

Although onychomycosis impacts individuals across all genders and ages, recent global trends show a burden among women of reproductive age who regularly use nail cosmetics^11^, as products like polishes, acrylic overlays, gel extensions, and artificial nails compromise nail plate integrity through repetitive trauma, aggressive filing, and chemical exposure, creating micro-fissures that may facilitate fungal invasion of the nail bed and matrix^12,13^. With 85–90% of women globally using enhancements, salons with shared unsterilized tools such as clippers and files increase potential fungal transmission risk^12,14,15^. The application/removal processes of gel polish and acrylic nail polishes involve mechanical trauma and acetone soaking which breach the nail barrier^16,17^, underscoring the urgency for improved sterilization and safer nail care practices.

Despite the clear biological plausibility linking nail cosmetic use to increased risk of onychomycosis, few studies in sub-Saharan Africa have specifically addressed this association in clinical settings^13,18,19^. Moreover, very little is known about how factors such as the frequency and type of cosmetic application, occupation, hygiene practices, or protective alternatives like henna use affect fungal nail infections in this context.

Therefore, this study aimed to determine the prevalence, ascertain the etiological agents, and identify associated factors of onychomycosis among women using nail cosmetics at Mbarara Regional Referral Hospital (MRRH) dermatology clinic in southwestern Uganda.

### Significance

This study addresses the pressing need to understand the prevalence of onychomycosis among women using nail cosmetics, a population increasingly at risk due to the popularity of manicure and pedicure services in communal salon settings that may facilitate fungal transmission. This will essentially help in public health interventions. By identifying the primary causative pathogens and associated factors, such as frequent nail cosmetic application and nail trauma, the research enables earlier diagnosis and tailored treatments, potentially reducing treatment failures and antifungal resistance while improving cost-effectiveness and patient outcomes. The findings underscore the importance of promoting safer nail care practices and highlight the necessity for targeted public health campaigns to lower onychomycosis incidence, alongside longitudinal studies to establish causality and long-term impacts, thereby filling critical knowledge gaps and enhancing management strategies for this chronic, quality-of-life-impacting condition.

## Methods

### Study design and period

This hospital-based cross-sectional study spanned January 2025 to March 2025 at MRRH skin clinic, having obtained approval from the Mbarara University of Science and Technology Research Ethics Committee (MUST-2024-1775) and MRRH administration. All procedures that were done in the study were carried out in accordance with the ethical standards of the institutional research committee and with the 1964 Helsinki Declaration and its later amendments or equivalents. Informed consent was obtained from all individual participants included in the study.

### Participant selection criteria

The study included adult female patients (≥ 18 years) using nail cosmetics within the past 12 months. Participants on current antifungal treatment for onychomycosis were excluded to avoid confounding results. Consecutive sampling enrolled all eligible, consenting participants until the sample size of 273 participants was met.

### Data collection procedures

A structured, pre-tested questionnaire aligned with the objectives of the study was administered and clinical findings recorded after examination.

### Specimen collection and laboratory diagnosis

Samples were collected aseptically: nails were cleaned with 70% alcohol, and clippings from the distal edge and subungual debris were obtained using sterile clippers and scalpels. Samples were examined using potassium hydroxide (KOH) microscopy (20% solution, 10–15-min incubation, examined at ×10 and ×40) and cultured on Sabouraud dextrose agar (SDA) with chloramphenicol, incubated at 25 °C for 14–21 days. Isolates were identified using colony morphology and lactophenol cotton blue staining with detailed notes on microscopic features.

### Data management and statistical analysis

Data were entered into Microsoft Excel 2020, cleaned, and analyzed in STATA v12.0. Prevalence was calculated with 95% confidence intervals (CIs). Logistic regression (univariate and multivariate) identified associated factors; odds ratios (ORs), 95% confidence intervals (CIs), and p-values were calculated using Wald’s test (*p* < 0.05 significant). Subgroup analyses explored age and occupation effects, with interaction terms tested.

## Results

A total of 273 participants were enrolled in the study.

### Demographic characteristics

The median age and Interquartile range (IQR) are shown in Table 1.

**Table 1:** Demographic characteristics of the study participants (n = 273).

	n	%
Median = 22 years (IQR 21–25)		
Age (Years)		
10–19	9	3.30
20–29	**230**	**84.25**
30–39	29	10.62
40–49	5	1.83
Education level		
Primary	13	4.76
Secondary	33	12.09
Tertiary	**227**	**83.15**
Occupation		
Students	**183**	**67.03**
Health workers	22	8.06
Sales & Retail	24	8.79
Service & Hospitality	17	6.23
Professional/Office work	17	6.23
Unemployed	10	3.66

### Prevalence of onychomycosis

Of the 273 women using nail cosmetics, 157 (57.5%, 95% CI) had positive cultures, distributed by demographics as detailed in Table 2.

**Table 2:** Distribution of onychomycosis cases by demographic characteristics. (n = 157).

	n	%
Age (Years)		
10–19	8	5.10
20–29	**127**	**80.89**
30–39	19	12.10
40–49	3	1.91
Occupation		
Students	**98**	**62.42**
Health workers	15	9.55
Sales & Retail	19	12.10
Service & Hospitality	11	7.01
Professional/Office work	11	7.01
Unemployed	3	1.91
Education level		
Primary	7	4.46
Secondary	22	14.01
Tertiary	**128**	**81.53**

### Etiology of onychomycosis

**Fig. 1:**
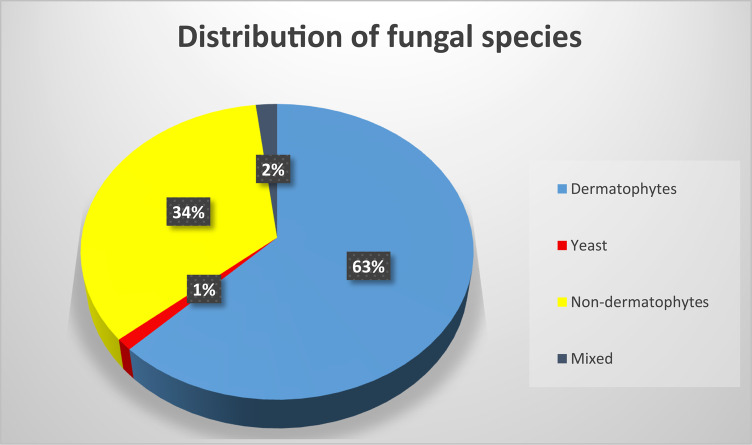
Distribution of isolated fungal species. A pie chart showing percentage distribution of fungal isolates from 157 positive cultures. Categories: Dermatophytes (blue, 62.4%), NDMs (yellow, 34.4%), Yeasts (red, 1.3%), Mixed (black, 2.0%). NDM = non-dermatophyte molds. Identified using lactophenol cotton blue (LPCB) staining on Sabouraud dextrose agar.

Dermatophytes predominated (62.4%), followed by NDMs (34.4%) and yeasts in 1.3% of cases (Fig. 1; Table 3).

**Table 3:** Isolated fungal species in onychomycosis cases (n = 157).

Fungal species isolated	n	%
Non-dermatophyte molds		
*Aspergillus flavus*	**16**	**10.2**
*Aspergillus nigra*	7	4.5
*Curvularia spp*	4	2.6
*Acremonium spp*	6	3.8
*Penicillium spp*	9	5.7
*Bipolaris spp*	3	1.9
*Fusarium spp*	6	3.8
*Rhizopus spp*	3	1.9
***Candida spp***	2	1.3
Dermatophytes		
*Trichophyton mentagrophytes*	**41**	**26.1**
*Trichophyton rubrum*	**21**	**13.4**
*Trichophyton tonsurans*	3	1.9
*Microsporum canis*	15	9.6
*Microsporum gypsum*	18	11.5
Mixed		
*Rhizopus, Microsporum gypsum*	1	0.6
*Rhizopus, T. mentagrophytes*	1	0.6
*T. mentagrophytes, Microsporum*	1	0.6
Total	**157**	**100.0**

### Associated factors

In univariate analysis, onychomycosis was significantly associated with Sales/Retail occupation, Frequent polish application (< 1 month) and nail trauma history, with henna having protective properties as shown in Tables 4 and 5.

**Table 4:** Univariate analysis of demographic factors associated with onychomycosis.

Demographic	Affected n = 157 (%)	Not Affected n = 116 (%)	OR	95% CI	*P*-value
Age					
10–19	8 (88.9)	1 (11.1)			
20–29	127 (55.2)	103 (44.8)	0.20	0.0189676–1.25239	0.08
30–39	19 (65.5)	10 (34.5)	0.23	0.0259138–2.17669	0.203
40–49	3 (60.0)	2 (40.0)	0.19	0.0120733–2.91190	0.232
Education level					
Primary	7 (53.8)	6 (46.2)			
Secondary	22 (66.7)	11 (33.3)	1.71	0.4631216–0.34558	0.420
Tertiary	128 (56.4)	99 (43.6)	1.11	0.3610375–3.40176	0.857
Occupation					
Students	98 (53.5)	85 (46.5)			
Health workers	15 (68.2)	7 (31.8)	1.86	0.7238385–4.77233	0.198
Sales & Retail	**19 (79.2)**	5 (20.8)	**3.37**	1.180122–9.20504	**0.023**
Service & Hospitality	11 (64.7)	6 (35.3)	1.59	0.5641404–0.482098	0.380
Professional/Office work	11 (64.7)	6 (35.3)	1.59	0.5641404–4.48209	0.380
Unemployed	3 (30.0)	7 (70.0)	0.37	0.0932042–1.48251	0.161

**Table 5:** Univariate analysis of other factors associated with onychomycosis.

Factors	Affected n = 157 (%)	Not Affected n = 116 (%)	OR	95% CI	*p*-Value
Cosmetic type					
Gel polish	69 (65.7)	36 (34.3)			
Regular Polish	25 (52.1)	23 (47.9)	0.57	0.2830221–1.1363	0.110
Henna	26 (49.1)	27 (50.9)	0.50	0.25642- .984405	**0.045**
Artificial	3 (60.00)	2 (40.0)	0.78	0.1250385- 4.8983	0.793
Multiple	34 (54.8)	28 (45.2)	0.63	0.3332909–1.2042	0.164
Frequency					
Frequent (< 1 month)	16 (76.2)	5 (23.8)			
Regular (1–2 months)	77 (61.1)	49 (38.9)	0.49	0.169092 -1.4	0.191
Rarely (> 2 months)	64 (50.8)	62 (49.2)	0.32	0.1114046—.934	**0.037**
Chronic illness					
Yes	15 (65.2)	8 (34.8)	1.43	0.5833897–3.4858	0.436
No	142 (56.8)	108 (43.2)			
History of nail trauma					
Yes	**76 (66.1)**	39 (33.9)	1.85	1.127702 -3.0430	**0.015**
No	81 (51.3)	77 (48.7)			
Dermatological illness					
Yes	7 (70.0)	3 (30.0)	0.57	0.1439323- 2.2486	0.416
No	150 (57.0)	113 (42.9)			

In the adjusted multivariate model, a history of nail trauma and sales/retail occupation were strong independent associated factors for onychomycosis, whereas applying nail cosmetics rarely (> 2 months interval) was significantly protective as shown in Table 6.

**Table 6:** Multivariate analysis and stepwise model for factors associated with onychomycosis.

Predictor	Odds ratio	P > z	[95% conf. interval]
History of trauma	1.98	0.008	1.192005–3.29252
Frequency: Rarely (> 2 months)	0.61	0.049	0.371068–0.998688
Occupation: Sales & Retail	3.37	0.022	1.193876–9.510104

## Discussion

### Prevalence of onychomycosis among nail cosmetic users

This study revealed a high prevalence of onychomycosis (57.5%) among women using nail cosmetics, underscoring the burden of fungal nail infections in this specific at-risk group. These findings align with a hospital-based study in Benue State, Nigeria, where 86.6% of participants with a history of manicure and pedicure were diagnosed with onychomycosis^18^. The lower prevalence observed in our study may reflect methodological differences; ours targeted women with history of nail cosmetic use in a general outpatient dermatology clinic, while the Nigerian study included individuals with overt signs of infection.

Artificial nail users in our cohort exhibited a 46.2% infection prevalence, a figure substantially lower than the 98.5% reported by Shemer et al. in a specialized dermatology referral center in Israel^13^. This contrast likely reflects sample bias; while Shemer’s cohort was selected for post-enhancement complications, our broader sampling captured a wider spectrum of all nail cosmetic users with less emphasis on clinical appearance. Nevertheless, both studies support the role of artificial nails, which can trap moisture and compromise the nail barrier, facilitating fungal colonization.

### Aetiology of onychomycosis

The predominant etiological agents were dermatophytes, particularly *Trichophyton mentagrophytes* (26.1%) and *Trichophyton rubrum* (13.4%), aligning with global epidemiological trends that identify these species as leading causes of onychomycosis due to their keratinolytic properties^1^. Non-dermatophyte molds (NDMs) accounted for 34.4% of cases, most commonly *Aspergillus flavus*. This significant presence of NDMs echoes findings from studies in warm, humid regions where such molds thrive^2,19^. The comparison between clinical isolates and environmental swabs is particularly instructive. Whereas salon studies in Nigeria detected only NDMs on surfaces^3,19^, our clinical sampling revealed a broader spectrum of fungal species. This reinforces the need for direct clinical diagnostics to accurately determine infection etiology rather than inferring from environmental contamination alone.

*Candida* species were uncommon in our study (1.27%) despite their documented prevalence in healthcare settings and among artificial nail users in developed countries^13,20^. This discrepancy may reflect environmental and occupational differences; our participants were primarily students and sales workers, not healthcare providers who are more frequently exposed to nosocomial *Candida* species^21^.

### Associated factors of onychomycosis

Mechanical or chemical nail trauma was associated with increased likelihood of infection (OR = 1.98, *p* = 0.008). This is consistent with findings from studies in Mexico and the U.S., which documented that trauma, whether from aggressive filing, cuticle manipulation, or contact with acetone-based removers, compromises the nail barrier and predisposes individuals to fungal invasion^6,18,22^. Artificial nail adhesives and gel polish removers often contain acrylates that weaken the keratin structure, predisposing the nail to infection^17,23^.

Occupation also played a significant role. Women working in sales and retail settings had 3.37-fold higher odds of developing onychomycosis, likely due to workplace grooming standards and expectations, more frequent cosmetic use, and higher reliance on salons with inconsistent sterilization protocols. This demonstrates how cosmetic and occupational practices interact to increase the risk of disease, a topic that merits more research and intervention.

The frequency of cosmetic application was another critical factor. Infrequent users (> 2 months interval) exhibited significantly lower risk (OR = 0.61), supporting the theory that repeated cycles of application and removal cumulatively damage the nail^11^. These findings suggest that encouraging periodic breaks from nail cosmetics could be a cost-effective strategy to mitigate fungal infection risk.

An unexpected but promising finding was the protective association of henna use (OR = 0.50). Henna (*Lawsonia inermis*) is a plant-based product with documented antifungal activity against dermatophytes and *Candida* species^24,25^. Its topical, non-invasive nature, combined with cultural acceptability in the African context positions it as a potential public health intervention especially in low-resource settings.

In southwestern Uganda, particularly in urban Mbarara, the rapid expansion of cosmetic nail trends, such as gel polishes, acrylic extensions, and frequent manicures/pedicures, has become a prominent cultural practice among young women and students, fueled by societal pressure to maintain a pristine appearance and conform to beauty standards. Many of these services are provided in informal salons with inconsistent sterilization protocols, utilization of shared tools (e.g., clippers and files), and limited infection-control training, potentially increasing the risk of fungal exposure through contaminated equipment and repetitive nail trauma. These local beauty practices and hygiene challenges may contribute to the observed high prevalence of onychomycosis in this demographic, although the cross-sectional nature of the study limits the ability to draw causal conclusions.

### Limitations

The study’s reliance on self-reported data for nail cosmetic application frequency and details about history of nail trauma may introduce recall bias, potentially skewing the association with onychomycosis risk and limiting the accuracy of frequency and trauma-related findings. The hospital-based sample limits generalizability beyond clinic attendees. Future longitudinal studies could confirm these associations and explore interventions.

## Conclusion

Onychomycosis was prevalent (57.5%) among Ugandan women using nail cosmetics, predominantly caused by dermatophytes and associated with modifiable factors such as nail trauma and frequent cosmetic use. Sales-related occupations showed higher risk, while henna use appeared protective.

### Recommendations

Incorporation of routine nail examinations especially for women by clinical personnel.

Educational outreach campaigns aimed at nail technicians and users of nail cosmetics for safer practices and further henna research as a protective alternative.

Non-traumatic techniques for removing nail polish and adhesives should be promoted.

## Data Availability

The datasets generated and analyzed during the current study are available from the corresponding author upon reasonable request.
